# Potential Role of APOBEC3 Family Proteins in SARS-CoV-2 Replication

**DOI:** 10.3390/v16071141

**Published:** 2024-07-16

**Authors:** MST Monira Begum, Ayub Bokani, Samiul Alam Rajib, Mohadeseh Soleimanpour, Yosuke Maeda, Kazuhisa Yoshimura, Yorifumi Satou, Diako Ebrahimi, Terumasa Ikeda

**Affiliations:** 1 Division of Molecular Virology and Genetics, Joint Research Center for Human Retrovirus Infection, Kumamoto University, Kumamoto 860-0811, Japan; 2 School of Engineering and Technology, CQ University, Sydney, NSW 2000, Australia; 3 Division of Genomics and Transcriptomics, Joint Research Center for Human Retrovirus Infection, Kumamoto University, Kumamoto 860-0811, Japan; 4 Texas Biomedical Research Institute, San Antonio, TX 78227, USA; 5 Department of Microbiology, Faculty of Life Sciences, Kumamoto University, Kumamoto 860-8556, Japan; 6 Department of Nursing, Kibi International University, Takahashi 716-8508, Japan; 7 Tokyo Metropolitan Institute of Public Health, Tokyo 169-0073, Japan

**Keywords:** APOBEC3 family proteins, SARS-CoV-2, deaminase-independent mechanism, THP-1

## Abstract

Severe acute respiratory syndrome coronavirus 2 (SARS-CoV-2) has acquired multiple mutations since its emergence. Analyses of the SARS-CoV-2 genomes from infected patients exhibit a bias toward C-to-U mutations, which are suggested to be caused by the apolipoprotein B mRNA editing enzyme polypeptide-like 3 (APOBEC3, A3) cytosine deaminase proteins. However, the role of A3 enzymes in SARS-CoV-2 replication remains unclear. To address this question, we investigated the effect of A3 family proteins on SARS-CoV-2 replication in the myeloid leukemia cell line THP-1 lacking *A3A* to *A3G* genes. The Wuhan, BA.1, and BA.5 variants had comparable viral replication in parent and *A3A*-to-*A3G*-null THP-1 cells stably expressing angiotensin-converting enzyme 2 (ACE2) protein. On the other hand, the replication and infectivity of these variants were abolished in *A3A*-to-*A3G*-null THP-1-ACE2 cells in a series of passage experiments over 20 days. In contrast to previous reports, we observed no evidence of A3-induced SARS-CoV-2 mutagenesis in the passage experiments. Furthermore, our analysis of a large number of publicly available SARS-CoV-2 genomes did not reveal conclusive evidence for A3-induced mutagenesis. Our studies suggest that A3 family proteins can positively contribute to SARS-CoV-2 replication; however, this effect is deaminase-independent.

## 1. Introduction

Severe acute respiratory syndrome coronavirus 2 (SARS-CoV-2) is responsible for coronavirus disease 2019 (COVID-19). Since the first cases of novel coronavirus infection were detected in Wuhan, Hubei Province, China, in December 2019 [1,2], it spread rapidly worldwide. The World Health Organization (WHO) declared COVID-19 a Public Health Emergency of International Concern (PHEIC) on 30 January 2020 [3] and announced the termination of PHEIC on 5 May 2023 [4]. However, the COVID-19 pandemic has continued.

The apolipoprotein B mRNA editing enzyme polypeptide-like 3 (APOBEC3, A3) family of proteins is composed of seven DNA cytosine deaminases (A3A, A3B, A3C, A3D, A3F, A3G, and A3H proteins) in humans (reviewed in [5,6,7,8]). These A3 proteins are involved in an innate host defense mechanism against parasitic DNA-based elements (reviewed in [8,9,10,11]). Retroviruses are susceptible to cytosine to uracil (C-to-U) deamination caused by A3 family proteins because they produce single-stranded cDNA intermediates that act as the substrate for these enzymes (reviewed in [5,6,7,8,12]). Notably, human immunodeficiency virus type 1 (HIV-1) is the best-characterized substrate for A3 family proteins. In primary CD4^+^ T cells, at least four A3 enzymes (A3D, A3F, A3G, and only stable A3H haplotype) restrict HIV-1 replication by deaminating viral cDNA intermediates and physically blocking reverse transcription [13,14,15,16,17,18,19,20,21,22]. A3 enzymes recognize specific dinucleotide motifs for deamination, such as 5′-CC for A3G or 5′-TC for other A3 enzymes at target cytosine bases (the target cytosine is underlined), which appear as 5′-AG or 5′-AA mutations in the genomic strand [15,17,23,24]. A3 protein-mediated mutations were observed in the genome of single-stranded DNA virus [Transfusion-transmitted virus (TTV)] (reviewed in [8,12]) and various double-stranded DNA viruses [Epstein-Barr virus (EBV), herpes simplex virus type 1 (HSV-1), alpha human papillomaviruses (α-HPV), and BK polyomavirus (BK PyV)] (reviewed in [8,11,12]). However, due to deaminase-independent mechanisms, the antiviral activity of A3 family proteins is not simply associated with its enzymatic activity [25,26,27,28].

Although A3 family proteins generally prefer single-stranded DNA for deamination, several reports demonstrated that certain A3 proteins induce C-to-U mutations in single-stranded RNA substrates [29,30,31,32,33,34]. In addition, A3 protein-mediated mutations have been reported in the human coronavirus NL63 (HCoV-NL63) genome [35]. However, whether A3-induced mutations are associated with antiviral activity against RNA viruses remains unclear [35,36,37].

SARS-CoV-2 has been evolving continually since its emergence in late 2019 [38,39,40,41,42,43,44]. C-to-U mutations are among the most frequent mutations accumulated in the SARS-CoV-2 genome [39,40,41,43,44]. This has been speculated to be due to the deaminase activity of APOBEC proteins. These mutations are thought to produce viruses that are more infectious and evade adaptive immunity. Recently, it has been reported that A3A protein is the potential source of C-to-U mutations in the SARS-CoV-2 genome [40]. Another in vitro study focusing on APOBEC1 (A1), A3A, and A3G proteins suggested that A1 and A3A proteins (and, to a much lesser extent, A3G protein) have the capacity to mutate the SARS-CoV-2 genome; however, these mutations do not impact viral replication [45]. Unexpectedly, the expression of these editing enzymes promoted SARS-CoV-2 replication and propagation [45]. The authors suggested that APOBEC-induced mutations may provide a fitness advantage. Despite these illuminating studies, the role of A3 family proteins in SARS-CoV-2 replication is yet to be fully determined.

We have previously reported that A3H encoded in THP-1 is an unstable haplotype and not involved in the restriction to HIV-1 [21]. Further, we created the myeloid leukemia cell line THP-1 lacking *A3A*, *A3B*, *A3C*, *A3D*, *A3F*, and *A3G* genes (*A3A*-to-*A3G*-null) and characterized the susceptibility of *A3A*-to-*A3G*-null THP-1 cells to HIV-1 infection [21]. Importantly, these cell lines completely abolished restriction activity against Vif-deficient HIV-1 [21]. Therefore, we exploited the *A3A*-to-*A3G*-null THP-1 cells to create a version stably expressing angiotensin-converting enzyme 2 (ACE2) protein and examined the effect of A3 family proteins on SARS-CoV-2 replication.

## 2. Results

### 2.1. Creation of THP-1 and A3A-to-A3G-Null THP-1 Cells Stably Expressing ACE2 Protein

**Figure 1:**
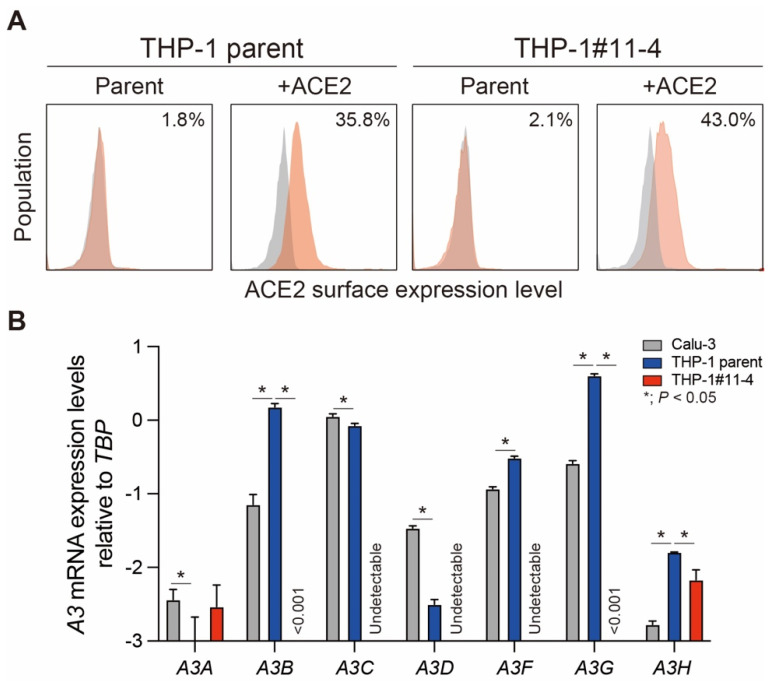
Validation of expression levels for ACE2 protein and *A3* mRNAs in THP-1 parent and *A3A*-to-*A3G*-null THP-1 cells. (**A**) ACE2 protein expression levels on the surface of THP-1 parent and THP-1#11-4 (*A3A*-to-*A3G*-null THP-1) cells. The *ACE2* gene was introduced by a retroviral vector, and the expression levels of the surface ACE2 protein were detected by an anti-ACE2 polyclonal antibody (red). The number in each graph shows the percentage of ACE2^+^ cells compared to those stained by isotype control (gray). (**B**) *A3* mRNA expression levels in Calu-3 (gray), THP-1-ACE2 (blue), and THP-1-ACE2#11-4 (red) cells. *A3* mRNA expression levels were quantified by RT-qPCR and normalized to *TBP* mRNA levels. Each bar represents the average of three independent experiments with Standard deviation (SD). Statistical significance was determined using the two-sided unpaired *t*-test. *, *p* < 0.05 compared to THP-1 parent cells.

To address whether A3 family proteins are associated with SARS-CoV-2 mutation and restriction, we introduced the *ACE2* gene into THP-1 parent cells and their derivatives lacking the expression of A3A to A3G proteins (THP-1#11-4). As shown in Figure 1A, ACE2 protein expression was not detected in THP-1 parent and THP-1#11-4 cells. However, 36% of THP-1 parental cells and 43% of THP-1#11-4 cells showed ACE2 protein expression after transduction. Next, *A3* mRNA expression levels in the lung epithelial cell line, Calu-3 and ACE2-transduced THP-1 cells were quantified by RT-qPCR (Figure 1B). Compared to Calu-3 cells, the expression levels of *A3B*, *A3F*, *A3G*, and *A3H* mRNAs were significantly higher in THP-1-ACE2 cells, but those of *A3A*, *A3C*, and *A3D* mRNAs were lower (Figure 1B). In THP-1-ACE2#11-4 cells, except for a minor difference in the *A3H* mRNA expression, *A3A* to *A3G* mRNA expression levels remain consistent with the previous observation [21] **(**Figure 1B). These data indicate that THP-1#11-4 cells stably express ACE2 protein and still fail to express the functional versions of any A3 family proteins.

### 2.2. Effect of A3 Family Proteins on SARS-CoV-2 Replication

**Figure 2:**
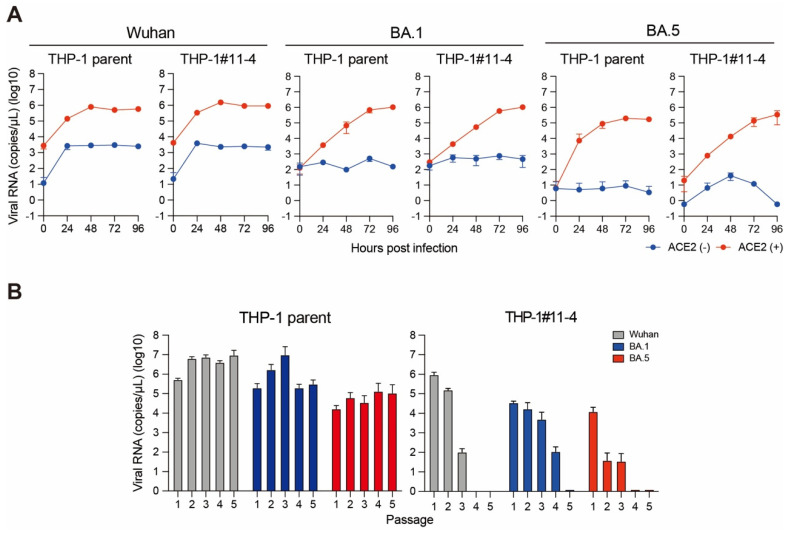
SARS-CoV-2 replication in THP-1 parent and *A3A*-to-*A3G*-null THP-1 cells. (**A**) Replication kinetics of the Wuhan, BA.1, and BA.5 variants produced from THP-1 parent and THP-1#11-4 (*A3A*-to-*A3G*-null THP-1) cells without (blue line) or with (red line) ACE2 protein expression. The SARS-CoV-2 *N* gene was quantified by RT-qPCR to monitor the viral RNA copy number across the indicated time points. Each timepoint represents the average of four independent experiments with SD. (**B**) Passage experiments. The SARS-CoV-2 *N* gene in the cell culture supernatants produced from THP-1-ACE2 or THP-1-ACE2#11-4 (*A3A*-to-*A3G*-null THP-1) cells at 96 h postinfection of each passage were quantified by RT-qPCR to monitor the viral RNA copy number of the Wuhan (gray), BA.1 (blue), and BA.5 (red) variants. Each bar represents the average of three independent experiments with SD.

We next investigated viral replication in THP-1 parent and THP-1#11-4 cells stably expressing ACE2 protein. We did not observe viral (Wuhan, BA.1, and BA.5) replication in cells lacking ACE2 protein expression, indicating that ACE2 is required for SARS-CoV-2 replication in THP-1 cells (Figure 2A). However, the replication of these variants was comparable between THP-1 parent and THP-1#11-4 cells during the 96-h time course (Figure 2A). The viral replication assay with the same time course was repeated until passage 5 (~20 days). In the THP-1 parent, the Wuhan, BA.1, and BA.5 variants showed continuous viral replication until passage 5 (Figure 2B). Surprisingly, the lack of *A3A* to *A3G* genes in THP-1 diminished the replication of all variants, and their viral RNAs became undetectable in passage 5 (Figure 2B). These data suggest that A3A to A3G proteins may be associated with long-term (~20 days) SARS-CoV-2 replication in THP-1 cells.

### 2.3. Effect of A3 Family Proteins on SARS-CoV-2 Infectivity

**Figure 3:**
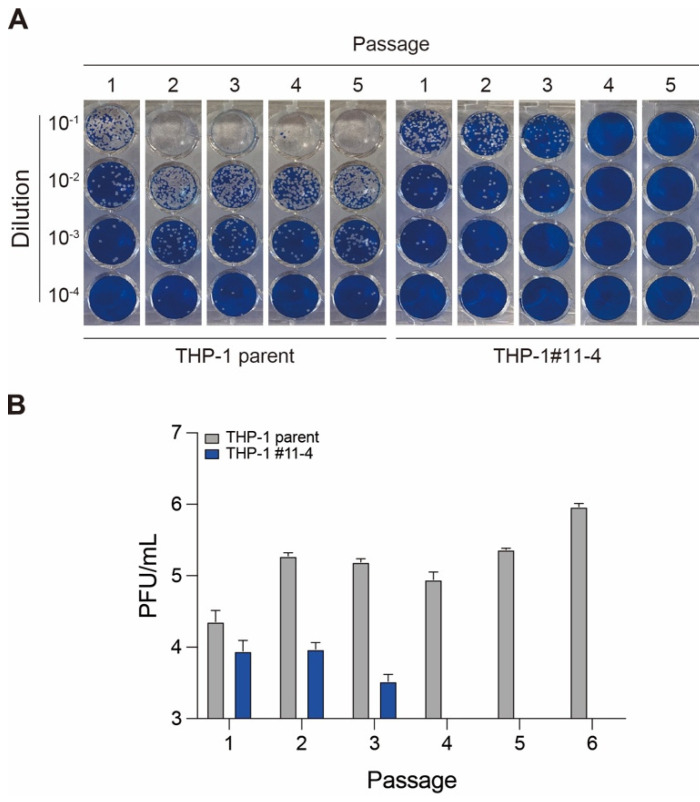
SARS-CoV-2 infectivity produced from THP-1 parent and *A3A*-to-*A3G*-null THP-1 cells during passage experiments. (**A**) Representative pictures of plaque assay. Cell culture supernatants obtained from the passage experiments for the Wuhan variant were also used for plaque assay with serial 10-times dilution. (**B**) PFU/mL of the Wuhan variant produced from THP-1-ACE2 (gray) or THP-1-ACE2#11-4 (*A3A*-to-*A3G*-null THP-1) (blue) cells at 96 h postinfection during passage experiments.

As mentioned above, the viral RNA of all variants tested became undetectable by 20 days postinfection, in contrast to that observed in THP-1 parent cells (Figure 2B). To know whether the Wuhan variant obtained from each passage was infectious, we performed a plaque assay in VeroE6/TMPRSS2 cells (Figure 3). Consistent with viral replication results (Figure 2B), the number of PFU obtained from the Wuhan variant infection in THP-1 parent cells was increased during the passage experiments (Figure 3). However, *A3A*-to-*A3G* gene disruption caused a decrease in viral infectivity, and it finally became undetectable (Figure 3). These data suggest that A3A to A3G proteins may contribute positively to the production of SARS-CoV-2 infectious particles from THP-1 cells.

### 2.4. Effect of A3 Proteins on SARS-CoV-2 Mutagenesis

**Figure 4:**
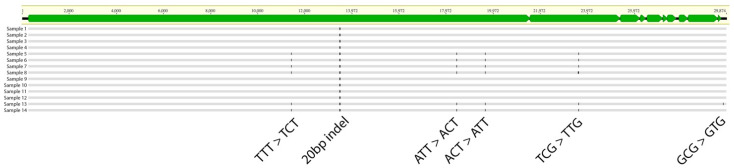
Analysis of mutations in SARS-CoV-2 genomes produced from THP-1 parent and *A3A*-to-*A3G*-null THP-1 cells during passage experiments. SARS-CoV-2 genomic RNA was isolated and subjected to WGS. Sample 1 to 4: Wuhan variant from passage 1 of THP-1-ACE2 cells. Sample 5 to 8: Wuhan variant from passage 5 of THP-1-ACE2 cells. Sample 9 to 12: Wuhan variant from passage 1 of THP-1-ACE2#11-4 (*A3A*-to-*A3G*-null THP-1) cells. Sample 13 and 14: Wuhan variant from passage 3 of THP-1-ACE2#11-4 cells. Green boxes on the top show each SARS-CoV-2 *ORF* gene with nucleotide position.

**Figure 5:**
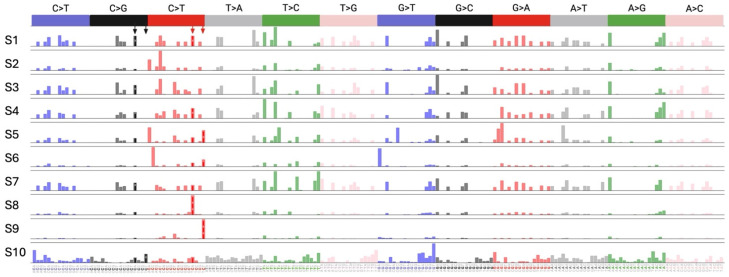
Analysis of mutational signatures in publicly available SARS-CoV-2 genomes. All possible 192 mutation types (NnN-to-NmN where n mutates to m and N:A/C/G/T) were quantified in a total of 38,830 whole-genome SARS-CoV-2 sequences sampled between the years 2019 and 2022 and reported in NCBI. These mutation counts were organized in a data matrix of (38,830 by 192) and used as input for analysis using the NMF method. Models with up to 20 components were built. Only a model with 10 components is shown for simplicity. None of the models showed signatures closely related to SBS2 and SBS13. Positions of the four major C>T and C>G peaks in SBS2 and SBS13 are shown by arrows.

Finally, we asked whether A3 proteins contribute to C-to-U mutations in the SARS-CoV-2 genome. We performed whole-genome sequencing (WGS) analysis for viral RNA isolated from each passage to address this question. Our analysis revealed mutations in seven positions, only two of which were C-to-T mutations, but none were in the TCA or TCT contexts, known as APOBEC targets (Figure 4). Additionally, there was no mutational burden difference between the cells with and without *A3A* to *A3G* genes (Figure 4). These results indicate that A3 proteins do not play a role in SARS-CoV-2 mutagenesis. To investigate whether the lack of A3-induced mutagenesis in SARS-CoV-2 is specific to THP-1 cells or is a general feature of SARS-CoV-2 infection, we conducted a bioinformatics analysis of 40,000 whole-genome SARS-CoV-2 sequences from NCBI. Our analysis involved the quantification of all possible 192 types of mutations followed by mutational signature deconvolution by the Non-negative Matrix Factorization (NMF). We investigated models with up to 20 mutational signatures and could not find any mutational signatures closely resembling single base substitution 2 (SBS2) [represented by TC(T/A)-to-TT(T/A)] or SBS13 [represented by TC(T/A)-to-TG(T/A)] as shown in Figure 5. However, we noted two signatures (S8 and S9), each represented by only one of the main SBS2 peaks (TCA-to-TTA in S8 and TCT-to-TTT in S9). Since such signatures containing only a single dominant peak have not been previously reported for any A3 enzymes, further studies are needed to provide evidence that these signatures are the footprint of A3 enzymes.

## 3. Discussion

The role of A3 enzymes in SARS-CoV-2 mutations has been implicated in several studies [39,40,41,43,44]; however, little is known about the functional relevance of A3 proteins in SARS-CoV-2 infection. In this study, we examined the effect of A3 proteins on SARS-CoV-2 replication by conducting infection in THP-1 cells lacking A3 enzymes. We showed that the Wuhan, BA.1, and BA.5 variants had comparable viral RNA production in THP-1-ACE2 parent and THP-1-ACE2 cells lacking the expression of A3A to A3G proteins during 96 h of the time course (Figure 2A). However, A3 family proteins affected SAR-CoV-2 RNA production in the passage experiments for up to 20 days (Figure 2B). Further, the plaque assay results showed that A3 family proteins might contribute to the production of infectious virus particles in THP-1 cells (Figure 3). Notably, the effect of A3 family proteins on SARS-CoV-2 replication is independent of C-to-U mutations (Figure 4). Taken together, our findings suggest that A3 family proteins may influence SARS-CoV-2 replication in a deaminase-independent manner.

C-to-U deamination by A3 family proteins is required to restrict HIV-1 [13,21,22,23,46,47,48,49,50]. However, deaminase-independent mechanisms also contribute to the anti-HIV-1 activity of A3 family proteins [14,21,48,49,51,52,53,54,55]. Indeed, deaminase-independent mechanisms are reported to be the predominant mode of viral restriction against many viruses [35,37,56,57,58,59]. Several mechanisms have been proposed for the deaminase-independent action. First, the binding of A3F and A3G proteins to HIV-1 genomic RNA blocks the elongation of reverse transcription directly [14,49,50,51,55]. Second, a direct interaction between the A3G protein and HIV-1 Reverse transcriptase causes the disruption of cDNA synthesis [20,60]. Third, an interesting notion is that the A3B protein promotes stress granule formation through a protein kinase R signaling pathway that mediates translational shutdown in cells infected with diverse RNA viruses, such as Sendai virus, Polio virus, and Sindbis virus [61]. These findings indicate that deaminase-independent mechanisms mediate the interaction of the A3 proteins with viral and non-viral proteins. Therefore, the range of innate defense mechanisms provided by A3 family proteins could potentially extend to many more viruses than those currently reported.

THP-1-ACE2 cells express *A3B*, *A3C*, *A3F*, and *A3G* mRNAs under normal cell culture conditions (Figure 1B). It has been shown that double-domain deaminases, A3B, A3F, and A3G proteins have the capacity to form high molecular mass ribonucleoprotein (HMM RNP) complexes [62,63,64,65,66,67,68]. Since HMM RNP complexes are composed of A3-binding RNAs, A3-binding proteins, and numerous cellular RNA-binding proteins [62,63,64,66,67,68,69,70], the interactions between the A3 proteins and viral and non-viral RNAs/proteins might play a role in supporting SARS-CoV-2 infectivity in THP-1 cells. Although it is controversial whether A3C protein, which is a single-domain deaminase, has the capacity to form HMM RNP complexes [65,71], this protein might also contribute to SARS-CoV-2 infectivity in THP-1. Nevertheless, additional studies are needed to fully understand the role of A3 proteins in SARS-CoV-2 infectivity.

THP-1 cells were derived from the blood of a male patient with acute monocytic leukemia [72]. In this study, we used THP-1 cells obtained from Dr. Cimarelli [73]. However, it is important to note that multiple sublines of THP-1 exist, and the potential impact of this intra-cell line heterogeneity on the results presented here requires further investigation. THP-1 cells do not express ACE2 and TMPRSS2 and are not natural targets of SARS-CoV-2 (Figure 1A and Figure 2A). It should be noted that adding TMPRSS2 expression to THP-1-ACE2 cells may give a different result that C-to-U mutations are induced on the SARS-CoV-2 genome. Therefore, the phenomenon observed in this study might be limited to THP-1 cells.

In summary, we found that A3 family proteins support SARS-CoV-2 replication in THP-1 independently of their enzymatic activity. Understanding the underlying mechanism may provide new insight into the interaction between A3 family proteins and coronaviruses. Further, A3 family proteins may be a potential therapeutic target for drug development to alleviate disease severity in respiratory diseases caused by coronaviruses.

## 4. Materials and Methods

### 4.1. Cell Lines and Culture Conditions

THP-1 cells were provided by Dr. Andrea Cimarelli (INSERM, Paris, France) [73]. The generation and characterization of THP-1 Δ*A3A*-to-*A3G*#11-4 has been reported previously [21]. THP-1 cells and their derivatives were maintained in RPMI1640 (Thermo Fisher Scientific, Waltham, MA, USA, Cat# C11875500BT) with 10% fetal bovine serum (FBS) (NICHIREI, Belrose, NSW, Australia, Cat#175012) and 1% penicillin/streptomycin (P/S) (Wako, Tokyo, Japan, Cat# 168-23191). GP2-293 cells (HEK293 expressing Moloney murine leukemia virus gag/pol protein; TAKARA, Kusatsu, Japan, Cat# 631530) were maintained in high glucose Dulbecco’s Modified Eagle Medium (DMEM, Wako, Cat# 044-29765) containing 10% FBS and 1% P/S. VeroE6/TMPRSS2 cells (VeroE6 cells stably expressing human TMPRSS2 protein; JCRB Cell Bank, JCRB1819) [74] were maintained in low glucose Dulbecco’s Modified Eagle Medium (DMEM, Wako, Cat# 041-29775) containing 10% FBS, G418 (1 mg/mL; Wako, Cat#070-06803) and 1% P/S (Wako, Cat# 168-23191). Calu-3 cells (ATCC, HTB-55) were maintained in EMEM (Wako, Cat#055-08975) containing 20% FBS and 1% P/S. All cells were maintained at 37 °C with 5% CO_2_.

### 4.2. Virus Preparation

SARS-CoV-2 Wuhan variant (strain SARS-CoV-2/Hu/DP/Kng/19-020, Genbank accession no. LC528232) [75,76] was provided by Drs. Tomohiko Takasaki and Jun-Ichi Sakuragi (Kanagawa Prefectural Institute of Public Health). SARS-CoV-2 Omicron BA.1 (strain TY38-873, GISAID ID: EPI_ISL_7418017) [76,77] variant was obtained from the National Institute of Infectious Diseases. BA.5 (strain TKYS14631; GISAID ID: EPI_ISL_12812500) [78,79,80] variants were provided by the Tokyo Metropolitan Institute of Public Health.

Virus propagation was performed as previously described [75,76,81,82,83]. Briefly, VeroE6/TMPRSS2 cells (5 × 10^6^ cells) were seeded in a T-75 flask the day before infection. The virus was diluted in virus dilution buffer [1M HEPES, DMEM (low glucose), Non- essential Amino acid (Gibco, Waltham, MA, USA Cat# 11140-050), 1% P/S], and the dilution buffer containing the virus was added to the flask after removing the initial medium. After 1 h of incubation at 37 °C, the supernatant was replaced with 15 mL of 2% FBS/DMEM (low glucose) and cell culture was continued to incubate at 37 °C until visible cytopathic effect (CPE) was clearly observed. Then, cell culture supernatant was collected, centrifuged at 300× *g* for 10 min and frozen at −80 °C as working virus stock. The titer of the prepared working virus was determined as the 50% tissue culture infectious dose (TCID_50_) [78,82,84]. The day before infection, VeroE6/TMPRSS2 cells (10,000 cells) were seeded in a 96-well plate and infected with serially diluted working virus stocks. The infected cells were incubated at 37 °C for 4 days, and CPEs were observed in the infected cells by a microscope. The value of TCID_50_/_mL_ was calculated by the Reed-Muench method [85].

### 4.3. A3 mRNA Quantification

Cells were harvested and washed with PBS twice. Then, total RNA was isolated by RNA Premium Kit (NIPPON Genetics, Cat# FG-81250), and cDNA was synthesized by Transcriptor Reverse Transcriptase (Roche, Basel, Switzerland, Cat# 03531287001) with random hexamer. RT-qPCR was performed using Power SYBR Green PCR Master Mix (Thermo Fisher Scientific, Cat# 4367659). Primers for each *A3* mRNA have been reported previously [86,87]. *A3A* forward: (5′-GAG AAG GGA CAA GCA CAT GG) and *A3A* reverse: (5′-TGG ATC CAT CAA GTG TCT GG). *A3B* forward: (5′-GAC CCT TTG GTC CTT CGA C) and *A3B* reverse: (5′-GCA CAG CCC CAG GAG AAG). *A3C* forward: (5′-AGC GCT TCA GAA AAG AGT GG) and *A3C* reverse: (5′-AAG TTT CGT TCC GAT CGT TG). *A3D* forward: (5′-ACC CAA ACG TCA GTC GAA TC) and *A3D* reverse: (5′-CAC ATT TCT GCG TGG TTC TC). *A3F* forward: (5′-CCG TTT GGA CGC AAA GAT) and *A3F* reverse: (5′-CCA GGT GAT CTG GAA ACA CTT). *A3G* forward: (5′-CCG AGG ACC CGA AGG TTA C) and *A3G* reverse: (5′-TCC AAC AGT GCT GAA ATT CG). *A3H* forward: (5′-AGC TGT GGC CAG AAG CAC) and *A3H* reverse: (5′-CGG AAT GTT TCG GCT GTT). *TATA-binding protein* (*TBP*) forward: (5′-CCC ATG ACT CCC ATG ACC) and *TBP* reverse: (5′-TTT ACA ACC AAG ATT CAC TGT GG). Fluorescent signals from resulting PCR products were acquired using a Thermal Cycler Dice Real Time System III (Takara). Finally, each *A3* mRNA expression level was represented as values normalized by *TBP* mRNA expression levels (Figure 1B).

### 4.4. ACE2 Transduction

pLV-EF1a-human ACE2-IRES-Puro [88] was used as a template to amplify human *ACE2* gene using primers, forward: (5′-NNN NNG TTA ACA CCA TGT CAA GCT CTT CCT GGC TCC TTC) and reverse: (5′-NNN NNC TCG AGC TAA AAG GAG GTC TGA ACA TCA TCA GTG). Then, the amplified human *ACE2* gene was inserted into the pMSCVneo retroviral vector (Takara, Cat# 634401) at HpaI and XhoI sites. The inserted human *ACE2* gene was Sanger sequenced (AZENTA), and the data were analyzed using Sequencher DNA sequence analysis software v5.5.6 (Gene Codes Corporation).

Retroviral transduction was performed as previously described [46,48,88]. VSV-G-pseudotyped virus expressing human ACE2 protein was generated by transfecting 4 µg of pMSCV-ACE2-neo plasmid and pVSV-G expression vector (Addgene, cat# 138479) using TransIT-LT1 reagent (Takara, Cat# MIR2306) into GP2-293 cells (3 × 10^6^ cells). Forty-eight hours later, supernatants were harvested, filtered (0.45 μm filters, Merck, Cat# SLHVR33RB), and subjected to ultracentrifugation at 22,000× *g* at 4 °C for 2 h. After resolving the viral pellets with 10% FBS/RPMI1640, the concentrated retrovirus was inoculated into THP-1 cells and their derivatives (1.5 × 10^5^ cells) and incubated at 37 °C. At 72 h posttransduction, the cells were selected by 1 mg/mL G418 (Wako, Cat#070-06803). G418-selected cells with relatively higher ACE2 expression were sorted by a FACS Aria II (BD Biosciences, San Jose, CA, USA) and expanded. After expansion, the expression level of surface ACE2 was verified by a FACS Canto II (BD Biosciences). A goat anti-ACE2 polyclonal antibody (R&D Systems, Minneapolis, MN, USA, Cat# AF933, 1:50) and an APC-conjugated donkey anti-goat IgG (R&D Systems, Cat# F0108, 1:50) were used for surface ACE2 staining (Figure 1A). Normal goat IgG (R&D Systems, Cat# AB-108-C, 1:100) was used as the negative control for this assay.

### 4.5. SARS-CoV-2 Infection

5 × 10^5^ parent and *A3A*-to-*A3G*-null THP-1 cells were seeded into a 24-well plate, inoculated with SARS-CoV-2 (5000 TCID_50_) and incubated at 37 °C for 1 h. After washing with phosphate-buffered saline (PBS), 1 mL of fresh cell culture medium was added. 15 μL of cell culture supernatant was harvested at the indicated timepoints and used for RT-qPCR to quantify the viral RNA copy number (see “RT-qPCR” section) (Figure 2A,B). For passage experiments, 10 µL of the cell culture supernatant at 96 h postinfection was transferred to the next target cells. The passage of the cell culture supernatant was repeated until passage 5.

### 4.6. RT-qPCR for SARS-CoV-2 RNA

RT-qPCR was performed as previously described [84,88,89,90,91,92]. Briefly, 5 μL of culture supernatant was mixed with 5 μL of 2 × RNA lysis buffer [2% Triton X-100 (Nacalai Tesque, Kyoto, Japan, Cat#12969-25), 50 mM KCl, 100mM Tris-HCl (pH 7.4), 40% glycerol, 0.8 U/μL recombinant RNase inhibitor (Takara, Cat# 2313A)] and incubated at room temperature for 10 min. 90 μL of RNase-Free Water was added, and then 2.5 μL of diluted sample was used for real-time RT-PCR according to the manufacturer’s protocol with One step TB green PrimeScript PLUS RT-PCR Kit (Takara, Cat# RR096A) and primers for *Nucleocapsid* (*N*) gene; Forward *N*, 5′-AGC CTC TTC TCG TTC CTC ATC-3′ and Reverse *N*, 5′-CCG CCA TTG CCA GCC ATT C-3′. The viral RNA copy number was standardized using a SARS-CoV-2 direct detection RT-qPCR kit (Takara, Cat# RC300A). Fluorescent signals from resulting PCR products were acquired using a Thermal Cycler Dice Real Time System III (Takara).

### 4.7. Plaque Assay

Plaque assay was performed as previously described [81,90,93,94]. One day before infection, 1 × 10^5^ VeroE6/TMPRSS2 cells were seeded into 24-well plates and infected with serial dilution of cell culture supernatants, including SARS-CoV-2 (10, 100, 1000, and 10,000-fold dilution, respectively) at 37 °C for 1 h. 3% FBS and 1.5% carboxymethyl cellulose (Wako, Cat# 039-1335) containing mounting solution was overlaid, followed by incubation at 37 °C. At 3 days postinfection, the cell culture medium was removed, and the cells were washed with PBS three times and fixed with 4% paraformaldehyde phosphate (Nacalai Tesque, Cat# 09154-85). The fixed cells were washed with tap water, dried, and stained with 0.1% methylene blue (Nacalai Tesque, Cat# 22412-14) in water for 30 min. The stained cells were washed with tap water and dried. Finally, the number of plaques was counted and indicated as plaque forming unit (PFU)/mL (Figure 3).

### 4.8. SARS-CoV-2 WGS

The WGS of the SARS-CoV-2 RNA genome was performed as previously described [95]. Briefly, cDNA synthesis, viral sequence enrichment, library amplification, and indexing were performed using the QIAseq DIRECT SARC-CoV-2 kit (QIAgen, Hilden, Germany, Cat# 333891) according to the manufacturer’s protocol. After multiplexing with QIASeq DIRECT UDI Set-A (QIAgen), a 25 µL library was prepared from each sample. The quality of the enriched libraries was evaluated by electrophoresis using the TapeStation 4150 system (Agilent Technologies, Santa Clara, CA, USA). The prepared libraries were subjected to sequencing using MiSeq reagent Micro and Nano Kits (Version 2, 300 cycles) in the MiSeq desktop sequencing system (Illumina, San Diego, CA, USA). The data analysis was done as previously performed [95].

### 4.9. SARS-CoV-2 Mutational Signature Analysis

We downloaded 40,000 whole-genome SARS-CoV-2 sequences from NCBI. These sequences were sampled between the years 2019 and 2022 and were highly diverse in terms of geographic distribution. A total of 38,830 of these sequences that had less than 5% insertion/deletion and/or non-A/C/G/T, were selected and aligned to the most common ancestor of SARS-CoV-2 as described by Kumar et al. [96]. The frequencies of all the possible 192 mutation types (NnN-to-NmN where n mutates to m and N:A/C/G/T) were quantified for each sequence. These mutation counts were organized in a data matrix of (38,830 by 192) and used as input for analysis using the Non-negative Matrix Factorization (NMF) method. NMF is a matrix decomposition method used routinely to deconstruct mutational signatures in cancer and viral genomes [97,98,99,100]. Here, NMF was used to investigate if the deconstruction of all SARS-CoV-2 sequences provides evidence for the existence of an APOBEC mutational signature similar to the known SBS2 and SBS13 signatures [97]. We built NMF models with up to 20 components to investigate the presence of SBS2 and SB13.

### 4.10. Statistical Analyses

GraphPad Prism software v8.4.3 was used for statistical analysis, including two-tailed unpaired *t*-tests (Figure 1B).

## 5. Conclusions

Our results suggest that A3 family proteins may contribute to SARS-CoV-2 replication in THP-1 cells. This effect of A3 family proteins on SARS-CoV-2 replication is independent of deaminase activity.

## Data Availability

Data are contained within the article.
